# The effect and safety of sleep interventions on suicidal thoughts and behavior – A systematic review and meta-analyses

**DOI:** 10.1016/j.sleepx.2025.100145

**Published:** 2025-06-18

**Authors:** Paula von Spreckelsen, Daan Schouten, Natasha G. Waslam, Patricia Katuin, Henriette D. Heering, Caroline Planting, Niki Antypa, Liia Kivelä, Marike Lancel, Lizanne J.S. Schweren

**Affiliations:** a 113 Suicide Prevention, Amsterdam, the Netherlands; b Dutch Institute for Forensic Psychiatry and Psychology (NIFP), the Netherlands; c Emergis, Specialized Mental Health Care, Kloetinge, the Netherlands; d Lived Experience Expert Team, Suicide Prevention Centre, the Netherlands; e Amsterdam University Medical Centre, Vrije Universiteit, the Netherlands; f GGZ inGeest Specialized Mental Health Care, Amsterdam, the Netherlands; g Department of Clinical Psychology, Institute of Psychology, Leiden University, Leiden, the Netherlands; h Centre of Expertise on Sleep and Psychiatry, GGZ Drenthe Mental Health Institute, Assen, the Netherlands; i Department of Clinical Psychology and Experimental Psychopathology, University of Groningen, Groningen, the Netherlands; j Forensic Psychiatric Hospital, GGZ Drenthe Mental Health Institute, Assen, the Netherlands

**Keywords:** Meta-analysis, Sleep intervention, Suicide, Psychotherapy, Pharmacotherapy

## Abstract

**Background:**

Sleep disturbances are a risk factor for suicidal ideation, suicide attempts and suicide. While recent findings suggest that improving sleep by psychotherapeutic sleep interventions exerts a positive effect on suicidality, there are also indications that certain sleep medications are associated with an increased risk for suicidality. Given these discrepancies, the current study systematically reviewed the effect of non-pharmacological and pharmacological sleep interventions on suicidality.

**Methods:**

A systematic literature search of various scientific databases was conducted (March 2025). Randomized controlled trials that examined the effect of sleep interventions on suicidality were searched for.

**Results:**

The search strategy resulted in 5037 unique articles. A total of *k* = 44 articles (*N* = 14965 participants) were included in the systematic review. The results indicate that sleep interventions, particularly psychotherapeutic sleep treatments (*g* = −0.17, [-0.26 to −0.08]), result in a small significant reduction of suicidal ideation. No conclusive evidence was found for the effect or safety of pharmacotherapy or light therapy targeting sleep disturbance on suicidality. The findings should be interpreted in light of the limited number of studies with often small sample sizes, methodological inconsistencies in monitoring suicidality as an adverse event, restrictions on the inclusion of people with suicidality, and reliance on suicidal ideation as an outcome measure of suicidality.

**Conclusion:**

Health care professionals are recommended to apply psychotherapeutic sleep interventions, such as cognitive behavioral therapy for insomnia, to reduce suicidal ideation in clients with disturbed sleep. When prescribing medications for disturbed sleep, we recommend professionals to closely monitor clients for potential adverse effects on suicidality.

## Highlights

• (Psychotherapeutic) Sleep interventions can reduce suicidal ideation.
• There is uncertainty about the effect and safety of pharmacological sleep interventions on suicidality.
• It is recommended that clinicians apply psychotherapeutic sleep interventions to target suicidality in clients with sleep disturbances.
• Clients receiving medication for disturbed sleep should be closely monitored for adverse effects on suicidality.

## 1 Introduction

Suicide constitutes a serious public health problem. Worldwide, more than 700,000 people die by suicide each year. For every completed suicide, many more people attempt it. Each loss is a tragedy that impacts families, communities, and entire countries, leaving lasting effects on those left behind [1]. We define suicidality as the range of thoughts, behaviors, and tendencies related to suicide including suicidal ideation (suicidal thoughts), suicide attempts and completed suicide. There are several risk factors for suicidality. Next to non-modifiable risk factors (such as age, gender, or a history of trauma), there are also modifiable risk factors to the experience of suicidality, including depression, substance abuse and sleep disturbances [e.g., 2]. Based on several meta-analyses, sleep disturbances have been identified as a risk factor for suicidal ideation, suicide attempts and death by suicide [e.g., 3,4]. A meta-analysis of cohort studies found that sleep disturbances are predictive of suicidality, demonstrating a 3.5-fold higher risk for suicide attempt and a 1.8-fold higher risk for completed suicide in people with sleep disturbances [2]. Another meta-analysis of longitudinal studies found that sleep disturbances were weakly predictive of suicidality, with insomnia and nightmares being the strongest predictors of suicidal ideation and suicide attempt respectively [3].

Sleep is fundamental for overall well-being as well as physical and mental health [4]. However, various factors such as stress, lifestyle changes, pain and psychiatric conditions can cause sleep problems [4]. Individuals experiencing disrupted sleep often endure various adverse effects, including daytime fatigue, mood disturbances and impaired cognitive function [[5], [6], [7], [8]]. Sleep disorders (or sleep-wake disorders) involve long-lasting problems with the quality, timing, and/or duration of sleep, which result in daytime distress and impairment in functioning [9]. While insomnia is the most common sleep disorder [10], other sleep-wake disorders include hypersomnia, parasomnias (such as nightmares), obstructive sleep apnea syndrome, narcolepsy, or restless leg syndrome. Sleep disorders are known to be a risk factor for other clinical conditions (e.g., anxiety disorders, depressive disorders [6,[11], [12], [13]]). There are several mechanisms that might underlie the relation between sleep disturbances and the increased risk for suicidality [14]. For example, there is often little social support and preventative measures available during the nighttime [15], making nocturnal wakefulness risky. Insufficient or inadequate sleep may also contribute to the downward spiral of negative thinking [16,17], unhelpful emotion regulation strategies [15], reduced executive function/problem solving [18] and can promote impulsive and aggressive behavior due to a decline of self-regulation resources [19,20].

There are various approaches to treat sleep disorders [21,22]. The most frequently used are cognitive behavioral therapy for insomnia (CBT-I) and pharmacological treatment. CBT-I is an evidence-based treatment that aims to improve sleep quality and efficiency by modifying maladaptive cognitions and behaviors contributing to chronic insomnia. It typically includes components such as sleep education, sleep hygiene, cognitive restructuring, stimulus control, relaxation techniques, sleep restriction, and sleep scheduling. Furthermore, there are treatments for specific sleep disorders other than insomnia, such as continuous positive airway pressure (CPAP) for obstructive sleep apnea or imagery rehearsal therapy (IRT) and eye movement desensitization and reprocessing (EMDR) for the treatment of nightmares. Chronobiological treatments, like light therapy, have also been used to improve sleep disturbance [22]. Generally, it is recommended to first address insomnia with cognitive behavioral therapy before considering pharmacotherapy [22,23]. The most frequently prescribed medicines for insomnia, commonly referred to as sleep medications, are benzodiazepines and melatonin, but there is a wide variety of other sleep medications, such as dual orexin receptor antagonists (DORA's [24]) or Z-drugs (benzodiazepine-like drugs with mainly hypnotic effects and less anxiolytic, anti-epileptic and muscle relaxant effects). The advantage of pharmacotherapy lies in its rapid effect and often low cost. However, there are several drawbacks associated with the pharmacological approach to insomnia [22,23,25]. Although sleep duration and sleep continuity often improve, there can be a negative impact on both deep sleep and rapid eye movement (REM) sleep. Additionally, there is a risk of side effects, such as daytime drowsiness, the development of tolerance and dependence, or driving impairments (see for example [26,27]). It is not recommended to use sleep medications for the long-term management of insomnia, but instead to limit treatment duration to a short term (a maximum of 3–4 weeks). On the other side of the pharmacological spectrum, wake-promoting agents like armodafinil or solriamfetol can be used in the management of sleep disorders such as narcolepsy. Side effects of stimulant medications may include increased feelings of anxiety and risk of addiction [28,29].

Knowing that disturbed sleep can increase the risk for suicidality and that there are effective interventions for sleep disorders, one would expect that treatment of sleep disturbances reduces the risk for suicidality. However, so far it is not completely clear to what extent the treatment of sleep problems influences suicidality. There are indications that CBT-I may contribute to a reduction in suicidality. In one of the first trials examining suicidal ideation as an outcome, Manber and colleagues [30] reported data from 301 adults who participated in seven 90-min group CBT-I sessions, finding that 23 % of patients endorsed suicidal ideation before treatment, with this rate decreasing to 10 % post-treatment. A large-scale evaluation found that CBT-I was associated with reduced suicidal ideation among veterans, even after accounting for changes in depression severity, suggesting a direct link between improved insomnia and reduced suicide risk [31]. On the other hand, a large-scale analysis of U.S. survey data (2015–2018) revealed that prescription of multiple sleep medications, including Z-drugs, trazodone, and sedative benzodiazepines, are significantly associated with increased risks of suicidal ideation, planning, and attempts, even after adjusting for demographic and mental health factors [32]. These findings highlight the need for a systematic examination of the effect of both pharmacological and non-pharmacological sleep interventions on suicidality. To the best of our knowledge, no systematic review has yet been conducted on randomized controlled trials (RCTs) investigating the effects of treatments for various sleep disorders.

In the current project, we conducted a systematic review to provide insight into the extent to which the treatment of sleep disorders can affect suicidality. With this systematic investigation we aim to provide answers to the following research questions: (1) What is the overall effect of treating disturbed sleep on suicidality? and (2) What is the effect and safety of different types of sleep interventions on suicidality? Conducting a systematic review on the effect of treating disturbed sleep on suicidal thoughts and behavior is crucial for several reasons. While existing studies have evaluated specific sleep treatments for suicidality, such as the previously mentioned CBT-I or pharmacological interventions, there is a need for a comprehensive overview. Healthcare providers often face the challenge of integrating diverse findings from different studies when making treatment decisions. A systematic review and meta-analysis that synthesizes the evidence across various sleep disturbances and treatment options would provide a useful resource, offering an overview of the overall impact of sleep disorder treatment on suicidal thoughts and behavior which may guide more effective and informed clinical practices. Additionally, by examining both various sleep disorders and different treatment approaches together, we may gain deeper insights into the mechanisms underlying the relationship between sleep and suicidality.

## 2 Method

The current project was conducted in accordance with the PRISMA guidelines [36].

### 2.1 Literature search and study inclusion

The literature search was conducted on the June 4, 2023 and included all articles published before this date, using the following terms: suicide (and related terms), sleep (and related terms), randomized controlled trial (and related terms). The literature search was repeated with the same search terms on March 21, 2025. The following databases were searched for eligible articles: PubMed, Embase (including preprint archives biorxiv and medrxiv), APA PsycInfo, Web of Science, Cochrane Library - CENTRAL, and Scopus. See appendix A for the search strings used per database that were created by the medical information specialist. After removal of duplicates, articles were screened in the program Rayyan [34]. As a first step, the titles and abstracts of articles were screened by three evaluators, with each article being screened by at least two different evaluators. As a second step, the full texts of the remaining articles were screened by two evaluators. Disagreements between the evaluators were discussed among the evaluators. In cases of remaining uncertainty, the respective article(s) were discussed with the other co-authors. Further potential articles were searched from reference lists of relevant articles (including previously conducted systematic reviews/meta-analyses) and by searching the grey literature (Google Scholar search; searching for articles that cited key articles; searching other preprint databases such as psyarxiv).

#### 2.1.1 Eligibility criteria

We included peer-reviewed articles with RCT designs, which examined the difference between an intervention that targets sleep (i.e., sleep needed to be described as the mechanism of action and needed to be measured as an outcome) compared to any control condition on the outcome of suicidality. Suicidality included suicidal ideation/thoughts/plans, suicide attempts or suicide as a primary/secondary/adverse outcome using any assessment tool. Articles written in English, Dutch, German, French or Italian were included and there were no restrictions based on sample characteristics (e.g., depressed patients, insomnia patients), study setting (e.g., online, offline) or on article publication date. Records were excluded in case the article was not accessible (after contacting authors), or did not report the relevant information. Double publications and trial protocols were excluded. Lastly, articles published in predatory journals and in which the co-authors considered reporting to be suspicious, were excluded.

### 2.2 Risk of bias

The risk of bias of included articles was independently assessed by three co-authors according to the Revised Cochrane risk of-bias tool for randomized trials (version March 15, 2019 [35]). In case of disagreements, consensus was reached in discussion between the co-authors.

### 2.3 Data extraction

Two researchers extracted the following information from all included studies: first author, publication year, number of participants (in total and per group), mean age/age range of participants, percentage of females, setting, recruitment strategy, diagnostic status of participants, inclusion criteria, exclusion criteria, type of sleep intervention, type of control condition, type of outcome (score for suicidal ideation and/or number of suicide attempts and/or death by suicide; score for sleep disturbance), effect size and any other clinical information deemed important by the researcher.

### 2.4 Data analysis

Studies were categorized as ‘*efficacy studies’* when they examined the change from pre-intervention to post-intervention suicidality scores in the experimental vs. the control group. Studies that reported suicidality as an adverse outcome, meaning that they monitored and/or reported cases with emerging suicidality during the study in experimental and control group(s), were categorized as ‘*safety studies’*. For efficacy studies with the outcome suicidal ideation, SMDs (standard mean differences) were calculated based on reported means and standard deviations. In case raw means and standard deviations were not reported, reported information (e.g., regression coefficients) were converted according to the guidelines provided by Harrer and colleagues [36] to a pre-calculated SMD. If reported information was not suitable to be converted, the authors of the respective studies were contacted with a request for providing the raw means and standard deviations.

Meta-analyses were performed in case there was a sufficient number (at least 4) of studies per type of outcome measure (suicidal ideation vs. attempts vs. suicides) and, in the context of the second research question, per type of intervention (psychotherapeutic interventions; medication types; other interventions). Studies that could not be included in meta-analyses (e.g., due to incompatible outcome type/data format such as reporting suicidal ideation in dichotomous format), were described narratively in the section corresponding to the type of intervention they utilized (section 3.2.2). All meta-analyses were performed in R (R version 4.3.2 [37]) using the following packages: meta (version 7.0–0 [38]), metafor (version 4.4.0 [39]), metapsyTools (version 1.0.11 [40]), esc (version 0.5.1 [41]), metaSEM (version 1.3.1), robvis (version 0.3.0.900 [42]), following guidelines provided by Harrer and colleagues [36]. When studies provided more than one effect size (e.g. due to several study arms or outcome assessments), a clustering vector was specified to identify which effect sizes came from the same study, resulting in a three-level meta-analysis model.

For the first research question, the effects of all efficacy studies on the outcome measures suicidality and sleep disturbance were examined using separate three-level random-effects meta-analyses. As a second step, a multivariate meta-analysis was planned to estimate the association between the suicidality and sleep disturbance effect sizes of the included efficacy studies. For the second research question, the effects of efficacy studies per sleep intervention type on the outcome measure suicidality were examined via three-level random-effects meta-analyses. Binary outcomes of cases of suicidality in the safety studies were examined via three-level random effects meta-analyses using the Mantel-Haenszel method [43,44] to calculate the pooled effect estimate. When the number of studies was insufficient to conduct a meta-analysis, findings of included studies were summarized narratively.

## 3 Results

### 3.1 Included studies

**Fig. 1:**
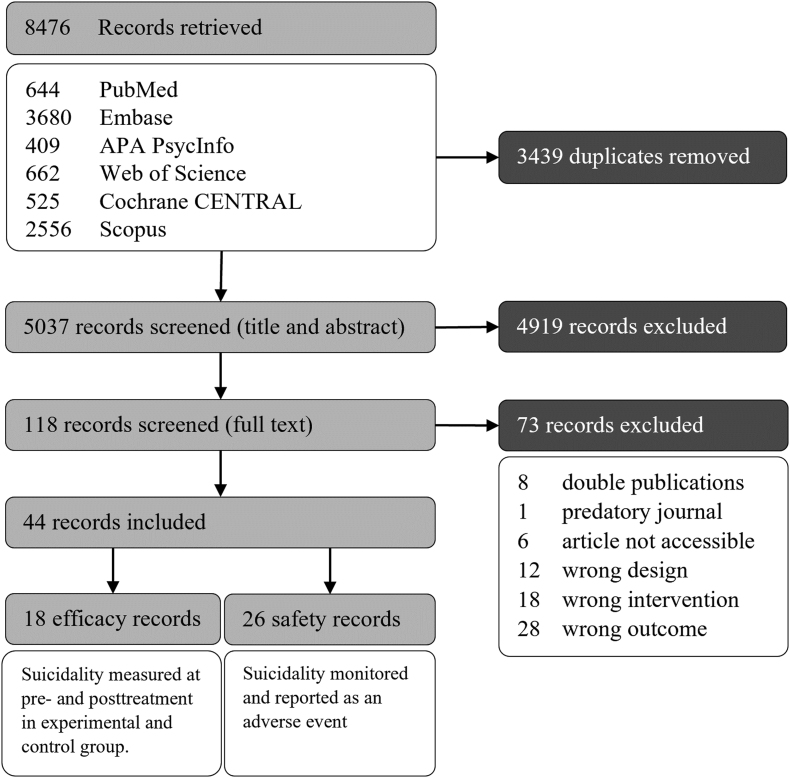
Flow diagram of the Study Inclusion.

A total of 18 efficacy studies (*n* = 2422) and 26 safety (*n* = 12543) studies were included in the systematic review. The flow diagram of study inclusion is depicted in Fig. 1. Table 1 displays an overview of all included efficacy and safety studies. Of the 18 efficacy studies, 13 employed psychotherapeutic interventions [CBT; 2 of the CBT-interventions included a light intervention component], 3 studied pharmacological therapies (2 studies with insomnia/sleep medication [Med-I], 1 with an off-label medication [Med-O]), and 2 studies examined the effects of light interventions [Light]. Of the 26 safety studies, 24 used pharmacotherapy (10 studies with insomnia/sleep medication [Med-I], 10 with medications against narcolepsy [Med-N], 4 with an off-label medication [Med-O]), and 2 studies examined light interventions [Light].

**Table 1:** Overview of studies included in the systematic review.

Author	Intervention category	Experimental Condition	Control Condition	Sample Size		% Women	Mean Age	Study Country	Disorder Inclusion	Suicidality Exclusion	Intervention duration		Suicidality Outcome	Measure of Suicidality
				N1	N2						Nr. Sessions	Nr. Weeks		
***Efficacy Studies***														
Batterham, 2017 [44]	CBT	digital intervention based on CBT-I	digital control program	574	575	73.5 %	43	au	insomnia; depression	yes (severe)	6	6	ideation	PSF
Chan, 2022 [46]	CBT	CBT-I	group therapy	45	45	67.4 %	20	aes	insomnia	yes (severe)	8	9	ideation	DSI-SS
			digital self-help	45										
Cromer, 2024 [47]	CBT	CBT for nightmares in children	waitlist	23	23	50 %	12.1	us	nightmares	yes (severe)	5	5	ideation & behavior (dichot.)	8 questions assessing suicidal thoughts and behavior
Crosby, 2025 [48]	CBT	Digital intervention based on CBT-I	digital control program	31	30	80 %	19.8	us	insomnia	no	1	1	ideation	DSI-SS
Ioannou, 2021 [49]	Light	sleep deprivation & daylight	sleep hygiene consultation + CAU	16	17	78.8 %	30.1	eu	depression	no	7	1	ideation	MADRS-S suicide item
Kalmbach, 2022 [50]	CBT	digital CBT-I	sleep education	60	66	71.4 %	41.37	us	insomnia; suicidality	no	6	6	ideation	QIDS-SR16 suicide item
Krystal, 2007 [51]	Med-I	3 mg eszopiclone + fluoxetine	placebo pill + fluoxetine	187	185	67.7 %	41.9	us	insomnia; depression	no	56	10	ideation	HAM-D
McCall, 2019 [52]	Med-I	6.25–12.5 mg zolpidem-CR + SSRI	placebo pill + SSRI	51	52	62 %	40.5	us	insomnia; depression	yes (severe)	56	8	ideation	BSSI
														C-SSRS
McCall, 2018 [53]	Med-O	prazosin + mood disorder meds	placebo pill + mood disorder medication	3	3	85 %	39.8	us	PTSD	yes (severe)	56	8	ideation	BSSI
														C-SSRS
Nazem, 2024 [54]	CBT	digital intervention based on CBT-I	sleep education	23	27	30 %	39.0	us	insomnia, suicidality	no	9	9	ideation	ASIQ
Pigeon, 2019 [55]	CBT	brief CBT-I	CAU for MDD and/or PTSD	24	26	20 %	nr	us	insomnia; depression, PTSD	yes (severe)	4	6	ideation	C-SSRS
Pigeon, 2017 [56]	CBT	brief CBT-I	sleep hygiene	13	14	11 %	58.5	us	insomnia; depression,	yes (severe)	4	5	ideation (dichot.)	C-SSRS
Pruiksma, 2020 [57]	CBT	exposure, relaxation, and rescripting therapy	minimal contact control	20	20	15 %	33.03	us	nightmare; trauma	yes (severe)	5	6	ideation	DSI-SS
Smagula, 2024 [58]	CBT (+Light)	transdiagnostic intervention for sleep and circadian dysfunction	CAU	40	57	53 %	46.5	us	mixed	yes (severe)	8	12	ideation (dichot.)	QIDS-SR16 suicide item
Sheaves, 2019 [59]	CBT	imagery-focused CBT for nightmares + CAU	CAU	12	12	42 %	nr	uk	nightmare; psychosis	no	4	4	ideation	BSSI
Volf, 2020 [60]	Light	dynamic lighting + CAU	static lighting + CAU	4	4	66.7 %	nr	eu	depression	yes (severe)	28	4	ideation	SIDAS
Waite, 2023 [61]	CBT (+Light)	pychological therapy for sleep problems + CAU	CAU	21	19	48 %	16.9	uk	Psychosis, insomnia	no	8	12	ideation	C-SSRS
Wu, 2023 [62]	CBT	mindfulness	waiting list	30	30	76.6 %	19.75	eas	suicidality	yes (severe)	8	8	ideation	BSSI
***Safety Studies***														
Ancoli-Israel, 2010 [63]	Med-I	2 mg eszopiclone	placebo pill	194	194	62.6	72	us	insomnia	no	84	12.0	suicide	nr
Bogan, 2022 [64]	Med-N	6 g ON-SXB	2 × 3 g SXB	28	28	54 %	39.6	us	None	yes (history)	2	1.0	ideation & behavior	nr
Brooks, 2019 [65]	Med-I	seltorexant (10/20/30/40 mg)	placebo pill	18	18	60 %	43	eu	depression; insomnia	yes (active)	4	4.0	ideation & behavior	C-SSRS
Czeisler, 2009 [66]	Med-N	150 mg armodafinil	placebo pill	123	122	46.7 %	39.6	oth	shift work disorder	no	42.4	12.0	ideation	nr
Dauvilliers, 2023 [67]	Med-N	30 mg danavorexton	placebo pill	17	17	58 %	31	oth	narcolepsy	yes	112	8	ideation & behavior	C-SSRS/spontaneous report
		90 mg danavorexton		20										
		180 mg danavorexton		19										
Dunlop, 2007 [68]	Med-N	200 mg modafinil + SSRI	placebo pill + SSRI	36	36	56 %	43.9	us	depression	yes (severe)	42	6.0	ideation	HAM-D & MADRS suicide item
During, 2023 [69]	Med-N	4.5 mg–9 mg SXB	Placebo pill	12	12	25 %	65.8	us	isolated/Parkinson's REM sleep behavior disorder	no	28	4	ideation	nr
Herring, 2020 [70]	Med-I	10 mg suvorexant	placebo pill	142	143	65 %	69.3	oth	insomnia; dementia	yes (severe)	28	7.0	ideation & behavior	C-SSRS
Herring, 2016 [71]	Med-I	suvorexant 15–20 mg	placebo pill	493	767	65 %	56	oth	insomnia	no	420	13.0	ideation & behavior	C-SSRS
		suvorexant 30–40 mg		770										
Kim, 2014 [72]	Med-O	300 mg quetiapine XR	600 mg lithium	12	17	55 %	36.1	eas	depression	no	56	8.0	behavior	nr
Krystal, 2022 [73]	Med-N	37.5–300 mg solriamfetol	placebo pill	355	119	52 %	45.6	us	OSA	yes (active)	84	12.0	ideation & behavior	C-SSRS
	Med-N	75–300 mg solriamfetol	placebo pill	177	59	67 %	36.3		narcolepsy					
Krystal, 2010 [74]	Med-N	200 mg armodafinil	placebo pill	124	124	53.8 %	49.5	us	OSA; depression	yes	84	12.0	ideation	QIDS-SR-16 item 12
Kunz, 2023 [75]	Med-I	10 mg daridorexant	placebo pill	142	128	71 %	58	oth	insomnia	yes	280	44.3	ideation & behavior	nr
		25 mg daridorexant		268										
		50 mg daridorexant		137										
		Ex-placebo 25 mg daridorexant		126										
Kushida, 2022 [76]	Med-N	9 g ON-SXB	placebo pill	107	105	67.2 %	31.2	oth	narcolepsy	yes	91	17.0	ideation	C-SSRS
Lieverse, 2011 [77]	Light	7,500lux bright pale blue light	50lux dim red light	42	47	65 %	69	eu	depression	no	21	6.0	suicide	nr
Lipschitz, 2023 [78]	Med-N	100/200 mg modafinil + SSRI	placebo pill + SSRI	6	4	70 %	48.4	us	bipolar disorder, sleep problems and/or cognitive impairment	yes (severe)	56	8	ideation & behavior	C-SSRS & BSSI
Locklear, 2013 [79]	Med-O	50–300 mg quetiapine XR	placebo pill	166	172	71.1 %	71.2	oth	depression	yes	63	11.0	behavior	nr
Loving, 2005 [80]	Light	1200 lux bright green light	10 lux dim red light	17	16	84.8 %	67.7	us	depression	no	28	21.0	behavior	nr
Mellman, 2022 [81]	Med-I	20 mg suvorexant	placebo pill	18	19	62.2 %	35.4	us	insomnia; trauma	no	42	7.0	ideation	C-SSRS
Mignot, 2022 [81]	Med-I	10 mg daridorexant	placebo pill	306	615	68 %	56	oth	insomnia	yes	91	40.0	ideation	C-SSRS
		25 mg daridorexant		616										
		50 mg daridorexant		308										
Nirogi, 2024 [83]	Med-N	2 mg samelisant	placebo pill	54	57	70.7 %	32.3	us	narcolepsy	no	14	2	ideation & behavior	C-SSRS
		4 mg samelisant		53										
Parmenter, 2024 [84]	Med-O	5.6 mg cyclobenzaprine	placebo pill	175	176	11.3 %	35.7	us	PTSD	yes (severe)	84	12	ideation & behavior	C-SSRS
Raskind, 2018 [85]	Med-O	12–20 mg prazosin	placebo pill	152	152	2.3 %	52	us	PTSD	yes (severe)	182	26.0	ideation	nr
Recourt, 2019 [86]	Med-I	20 mg seltorexant	placebo pill	22	12	34 %	40.6	eu	depression	no	19	8.5	ideation; suicide	C-SSRS
	Med-O	25 mg diphenhydramine		13										
Sangal, 2014 [87]	Med-I	1 or 2 mg eszopiclone (low)	placebo pill	163	160	36.2 %	11.4	us	insomnia; ADHD	no	84	12.0	ideation & behavior	C-SSRS
		2 or 3 mg eszopiclone (high)		159										
Uchimura, 2024 [88]	Med-I	50 mg daridorexant	25 mg daridorexant	102	52	55 %	54.4	eas	insomnia	yes (severe)	364	52	ideation & behavior	C-SSRS

*Note.* CBT = psychotherapeutic sleep intervention; Light = Light intervention; Med-I = medications against insomnia; Med-N = medications against narcolepsy; Med-O = off-label medication*;* CBT-I = cognitive behavioral therapy for insomnia; CAU = care as usual; PSF = Psychiatric Symptom Frequency Scale [88]; us = United States of America; eu = Europe; uk = United Kingdom; eas = East Asia; au = Australia & New Zealand; oth = other country & mix of countries; DSI-SS = Depressive Symptom Inventory Suicidality Subscale [90]; MADRS-S = Montgomery–Åsberg Depression Rating Scale (Self assessment) [91]; QIDS-SR16 = 16-Item Quick Inventory of Depressive Symptomatology [92]; ASIQ = Adult Suicidal Ideation Questionnaire [93] HAM-D = Hamilton Rating Scale for Depression [94]; BSSI = Beck's Scale for Suicide Ideation [95]; C-SSRS = Columbia-Suicide Severity Rating Scale [96]; SIDAS = suicidal ideation attributes scale [97].

### 3.2 Meta-analyses and descriptive results

#### 3.2.1 Research question 1: the effect of treating disordered sleep on suicidality

All efficacy studies assessed suicidal ideation as an outcome measure. Of the 18 efficacy studies, 9 reported the means and standard errors/deviations of suicidal ideation at pre- and posttreatment for both the control and intervention groups. Pre-calculated SMDs could be computed from the information provided in 5 studies (see guidelines by Harrer et al. [36]). Of the 4 studies which did not provide data that could be recalculated into SMDs (e.g., due to dichotomous data formats), authors were contacted and one responded with providing requested means and standard deviations. Therefore, the remaining 3 studies could not be included in the meta-analysis due to incompatible data format. This resulted in a total of 15 studies with 18 effect sizes to be included in the analyses. The majority of studies used the insomnia severity index (ISI [98]; 9 studies) and the remaining studies used the Pittsburgh Sleep Quality Index (PSQI [99]; 3 studies) to assess sleep disturbance.

**Fig. 2:**
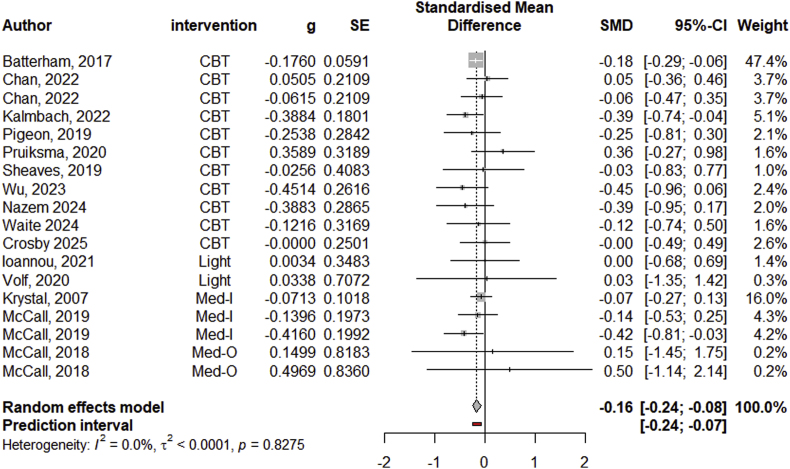
Forest Plot of all Efficacy Studies on Suicidal Ideation *Note.* CBT = psychotherapeutic sleep intervention; Light = light intervention; Med-I = medications against insomnia; Med-O = off-label medication.

When examining the efficacy-studies on the outcome sleep, we found that the included sleep interventions significantly decreased sleep disturbance in the experimental vs. control conditions (Hedge's *g* = −0.76, 95 % CI: 1.03 to −0.48; medium effect size; see B2 in Appendix B for more information). Examining the effect of all efficacy-studies on suicidality revealed that sleep interventions resulted in a small, but significant, decrease in suicidal ideation compared to control conditions (Hedge's *g* = −0.16, 95 % CI: 0.24 to −0.08). For more information, see the forest plot in Fig. 2. The funnel plot and publication bias correction models did not indicate presence of publication bias and the pooled effect remained statistically significant when excluding influential studies (see B1 in Appendix B). Due to model specification errors likely related to the differences in heterogeneity estimates between the suicidality outcome (*I*^*2*^ = 0 %) and the sleep outcome (*I*^*2*^ = 82 %), we were not able to conduct the multivariate meta-analysis to estimate the association between the suicidality effect sizes and sleep disturbance effect sizes.

#### 3.2.2 Research question 2: the effect and safety of different types of sleep interventions on suicidality

##### 3.2.2.1 Psychotherapeutic sleep interventions

**Fig. 3:**
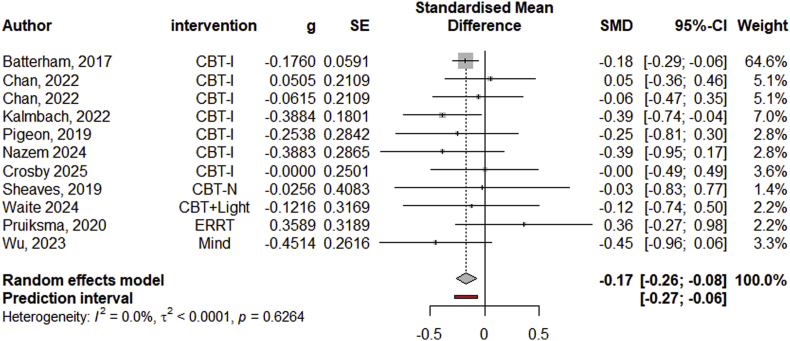
Forest Plot of Efficacy Studies with Psychotherapeutic Sleep Interventions on Suicidal Ideation *Note.* CBT-I = cognitive behavioral therapy for insomnia; CBT-N: cognitive behavioral therapy for nightmares; ERRT = exposure, relaxation, and rescripting therapy; Mind = mindfulness intervention; CBT + Light = psychotherapeutic sleep intervention with light component.

A total of 10 efficacy studies with 11 effect sizes were included in the meta-analysis to examine the effect of psychotherapeutic sleep interventions on suicidal ideation. The results indicated that psychotherapeutic sleep interventions had a small significant effect on decreasing suicidal ideation compared to control conditions (Hedge's *g* = −0.17, 95 % CI: 0.26 to −0.08; see forest plot in Fig. 3). The pooled effect did not remain statistically significant when excluding influential studies (see B3 in Appendix B). There were three efficacy studies that could not be included in the meta-analysis. One study examined the percentage of remission from suicidal ideation in the experimental and control groups ([56]) and reported 40 % remission in the experimental group (CBT-I) vs. 70 % remission in the control group. Another study reported cases with suicidal ideation before (experimental group: 12; control group: 21) and after (experimental group: 5; control group: 17) a transdiagnostic sleep intervention for sleep and circadian dysfunction [58]. Lastly, another study on CBT for nightmares in children reported remission of suicidal thoughts and behavior in 4 out of 5 participants in the experimental group, and 3 out of 5 in the control group, as well as the emergence of suicidal thoughts and behavior in 2 participants in the control group [47]. There were no safety studies employing psychotherapeutic sleep interventions.

##### 3.2.2.2 Pharmacological sleep interventions

###### 3.2.2.2.1 Medications for insomnia (sleep medication)

**Fig. 4:**
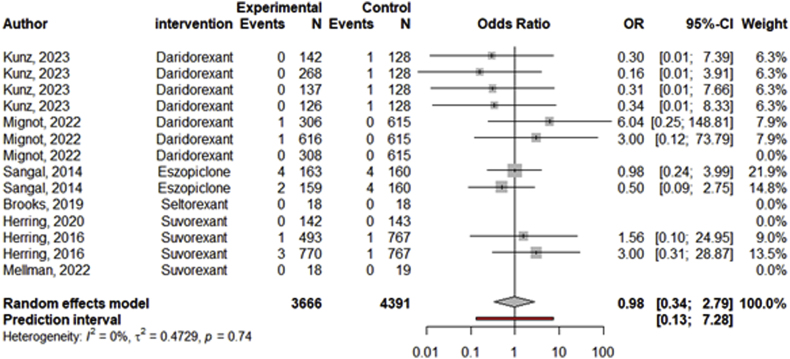
Forest Plot of Safety Studies with Medications Treating Insomnia on Suicidal Ideation Note. SXB(ON) = sodium oxybate (once-nightly).

Two efficacy studies examined the effect of sleep medications on changes in suicidal ideation. The examined medications were eszopiclone (*g* = −0.07; 95 % CI: 0.27 - 0.13 [51]) and zolpidem (*g* = −0.14; 95 % CI: 0.53 - 0.25 [suicidal ideation assessed with the BSSI], *g* = −0.42; 95 % CI: 0.80 to −0.03 [suicidal ideation assessed with the C-SSRS] [58]). The number of efficacy studies was not sufficient to conduct a meta-analysis. There were 7 safety studies with 14 effect sizes that reported monitoring cases of suicidal ideation in studies comparing sleep medications with placebo. The meta-analyses results did not indicate that the number of cases with suicidal ideation were significantly different between the control and experimental conditions (OR = 0.98, 95 % CI: 0.34–2.79; see forest plot in Fig. 4). Five of the 7 safety studies included in the analysis on suicidal ideation also monitored suicidal behavior but reported no cases of suicidal behavior [65,70,71,75,87]. One study comparing different dosages of daridoxant (50 mg vs. 25 mg), reported no cases of suicidal ideation or behavior in either group [88]. One study testing eszoplicone reported one suicide in the experimental group (*n* = 194) and no suicides in the control group (*n* = 194 [63]). Another study reported no suicides in either the seltorexant nor the placebo control group [86].

###### 3.2.2.2.2 Medications for narcolepsy

**Fig. 5:**
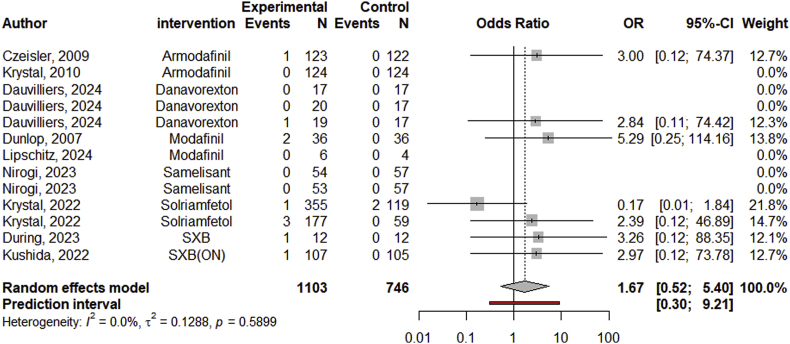
Forest plot of safety studies with medications treating narcolepsy on suicidal ideation.

We did not find any efficacy studies examining the effect of narcolepsy medications on changes in suicidality. There were 9 safety studies with 13 effect sizes that reported monitoring cases of suicidal ideation in studies testing narcolepsy medications (in any target group; see Table 1 for details) against placebo. The meta-analysis results do not indicate that cases of suicidal ideation were significantly different between the control and experimental conditions (pooled OR = 1.67, 95 % CI: 0.52–5.50; see forest plot in Fig. 5). Four of the 9 safety studies included in the analysis on suicidal ideation also monitored suicidal behavior but reported no cases of suicidal behavior [67,73,78,83]. One study comparing ON-SXB (Once-nightly sodium oxybate; *n* = 28) with SXB (sodium oxybate; *n* = 28) reported zero cases of suicidal ideation or behavior in both medication groups [64].

###### 3.2.2.2.3 Off-label medications

In total, 6 studies examined different off-label medications. One efficacy study examined the effect of prazosin as a medication against nightmares on changes in suicidal ideation (*g* = 0.14; 95 % CI: 1.47- 1.77 [suicidal ideation assessed with the BSSI], *g* = 0.50; 95 % CI: 1.12 – 2.12 [suicidal ideation assessed with the C-SSRS] [53]). Prazosin was also examined in one safety study [85], which found 15 cases of suicidal ideation in the experimental group (*n* = 152) and 23 cases of suicidal ideation in the control group (*n* = 152). One study in veterans with PTSD reported no cases of suicidal ideation or behavior occurring in the control group (*n* = 176) or experimental group (*n* = 175) receiving cyclobenzaprine [84]. Quetiapine was examined as a pharmacological intervention to improve sleep quality in two studies: Kim and colleagues [72] reported one suicide attempt in the experimental group (*n* = 12) vs. zero suicide attempts in the control group (*n* = 17) and Locklear and colleagues [79] reported one suicide attempt in the experimental group (*n* = 166) and one suicide attempt in the control group (*n* = 166). One study using diphenhydramine as a negative control (because this study also tested a sleep medication it is counted as MED-I in the description of included studies in section 3.1) reported one suicide in the diphenhydramine group (*n* = 13) vs. zero suicides in the placebo control group (*n* = 12 [86]).

##### 3.2.2.3 Sleep interventions using light exposure

In total, 4 studies examined sleep interventions using light exposure in relation to suicidality. Two efficacy studies examined the effect of light interventions on changes in suicidal ideation (*g* = 0.00; 95 % CI: 0.68 - 0.69 [49]; *g* = −0.72; 95 % CI: 2.16 - 0.72 [60]). Two additional efficacy studies examined a psychotherapeutic intervention that included a light component [58,61]. The results of these studies are described in the section on psychotherapeutic interventions (textual description & Fig. 3). No cases of suicide attempts [80] and no cases of suicides [77] were reported in two safety studies.

### 3.3 Risk of bias

Of the 18 included efficacy studies, 12 studies were rated to have a moderate risk of bias, 5 were rated to have a high risk of bias, and 1 was rated to have a low risk of bias. Of the 26 included safety studies, 4 were rated to have a low risk of bias, 19 as moderate risk of bias, and 3 as high risk of bias. See B3 in Appendix B for the risk of bias plots of the efficacy and safety studies.

## 4 Discussion

Sleep disturbances appear to be a risk factor for suicidal ideation, suicide attempts and death by suicide. In the current study, we conducted a systematic literature review and meta-analyses to examine the effect of pharmacological and non-pharmacological sleep interventions on suicidal thoughts and behavior. Screening through an initial number of 5037 retrieved unique articles resulted in a final sample of 44 articles (k = 18 efficacy studies; k = 26 safety studies) with in total *N* = 14965 participants. We found that sleep interventions, particularly psychotherapeutic sleep interventions, had a small positive effect on reducing suicidal ideation. No conclusive evidence was found for the effectiveness or safety of pharmaceutical sleep interventions or of interventions using light exposure.

### 4.1 The effect of improving disturbed sleep on suicidality

Examining all efficacy studies revealed that the included sleep treatments markedly reduced sleep disturbance. The sleep therapies also appeared to slightly, but significantly reduce suicidal ideation. Due to model specification errors, we could not test whether reductions in sleep disturbances were associated with reductions in suicidality. This may have been due to the deflated heterogeneity estimate of the suicidality outcome which likely resulted from the large error margins of the included studies and the high weight of one study relative to the weight of the other studies. Nonetheless, the findings do show that suicidal ideation can be effectively reduced when sleep is targeted. As such, the current review contributes to the existing literature on sleep disturbance acting as a contributing factor to the experience of suicidality [e.g., 3–6]. Sleep interventions therefore seem to be an appropriate treatment choice to target suicidality in patients with co-morbid sleep disturbances.

Although statistically significant, the treatment effect of sleep interventions on suicidality was relatively small. A recent systematic review of 8 articles examined the effect of sleep interventions on suicidal ideation and similarly reported a small, albeit not statistically significant, pooled effect size [100]. The small effect of the current analysis could be explained by the majority of the efficacy studies not specifically including people with (high levels of) suicidality, which might have restricted the observable treatment effects on suicidality. It could also reflect that sleep interventions may not be sufficient to markedly reduce suicidality, for example, when sleep disturbances are secondary to another mental health issue (e.g., depression, PTSD). In these contexts, targeting these underlying issues or suicidality directly may have additional benefits [e.g., 78,79]. Given the detrimental effects of disturbed sleep on mental and physical functioning [4], it nonetheless remains imperative to target sleep disturbances alongside other treatments to promote recovery and relapse prevention [e.g., 80–82].

### 4.2 The effect and safety of different types of sleep interventions on suicidality

#### 4.2.1 Psychotherapeutic sleep interventions

Concerning the effect of different sleep interventions, we found that psychotherapeutic sleep interventions had a small positive effect on reducing suicidal ideation. As the pooled effect size was no longer significant when the study with the highest weight was excluded from the analysis, the findings should be interpreted with caution. Of all included psychotherapeutic sleep intervention studies, the majority utilized CBT-I (6 studies), with the other studies utilizing a mix of other psychotherapeutic interventions (CBT for nightmares; Exposure, Relaxation, and Rescripting Therapy for nightmares; Mindfulness intervention to promote sleep quality). As such, CBT-I may be a promising psychotherapeutic intervention to reduce suicidal ideation alongside to improving sleep. Next to alleviating insomnia, CBT-I appears to effectively improve comorbid mental health conditions, such as depression, anxiety, PTSD and substance abuse disorders [101,102]. There is evidence suggesting that these ‘secondary’ treatment effects are mediated by improvements in insomnia as a result of CBT-I [103,103]. In line with that, one of the articles included in this review [50] found that insomnia remission due to CBT-I mediated reductions in suicidal ideation.

So far, the exact mechanisms of action of psychotherapeutic sleep interventions, such as CBT-I, on reducing suicidality remain unclear. CBT-I has been found to improve quality of life [104] and to be associated with increased energy levels, self-esteem, and functioning [30,105]. These effects of CBT-I on well-being may also contribute to improvements in suicidal ideation. In addition, by decreasing sleeplessness, CBT-I may also reduce the chance of escalation into suicidal behavior. For example, people would be less likely to get into a suicidal crisis at night, when there is little opportunity for support [15] and executive functions are reduced [18], or be less likely to engage in impulsive behavior that is associated with poor sleep [e.g., 22,23]. Unfortunately, none of the included studies utilizing psychotherapeutic sleep interventions examined suicidal behavior as an outcome. More research is needed to understand how psychotherapeutic sleep interventions reduce suicidality, and particularly how they affect suicidal behavior.

#### 4.2.2 Pharmacological sleep interventions

We also examined the effect and safety of different pharmacological sleep therapies on suicidality: medications targeting insomnia (eszopiclone, zolpidem, daridoxerant, seltorexant, suvorexant), medications targeting narcolepsy symptoms (armodafinil, modafinil, ON-SXB, solriamfetol) and off-label medications targeting nightmares or insomnia (prazosin, quetiapine, diphenhydramine). We could not test the effect of these medications on suicidality due to an insufficient number of efficacy studies. With regard to safety, neither medications treating insomnia nor medications for narcolepsy were found to be associated with an increased or decreased likelihood for the occurrence of suicidal ideation as an adverse event compared to placebo pills. Due to the rarity of events within these studies, the individual and pooled odds ratios had very large error margins, thus resulting in a low certainty of evidence. Too few safety medication studies reported on the occurrence of suicide attempts and suicides to allow drawing conclusions on the basis of these observations. We can thus conclude that little is known about the effect and safety of pharmacological sleep interventions on suicidality. Importantly, none of the pharmacological studies specifically included participants with suicidality, and around half (57.6 %) of the medication safety studies explicitly excluded participants with suicidality. In order to properly examine the effect/safety of (pharmacological) treatments on inherently rare events, particularly suicidal behavior, it is crucial that individuals with suicidality are not excluded from intervention trials. This means that there is substantial uncertainty about the possibility of suicidal adverse effects of pharmacological sleep interventions in people with (high levels of) suicidality.

There were striking differences in the monitoring and reporting of suicidality between the included medication safety studies. While some studies simply described participants self-reporting suicidal adverse effects, other studies monitored suicidality at each study visit. Within the latter group, not all studies described the monitoring instrument that was used and even fewer reported on pre-specified cut-off scores used for determining whether a suicidal adverse effect had occurred. Research indicates that inconsistencies in monitoring methods and underreporting of suicidality is a common problem in clinical trials [106,107]. This is particularly problematic in light of research finding associations between sleep medication use and increased risk of suicide [e.g., 30,91]. Pharmacological sleep interventions have several side effects, including reduced sleep quality, rebound insomnia, cognitive impairments, fatigue, somnolence, and the development of dependence [22,25,108]. Residual effects of pharmacotherapy for disturbed sleep further appear to be associated with impaired functioning (e.g., work impairment, difficulties in relationships) and decreased quality of life [109]. Such decreased life quality and impairments may contribute to increases in suicidality [110,111]. As such, occurrences of suicide attempts and suicides in medication groups should be seen as potentially concerning signals to be investigated further.

#### 4.2.3 Other sleep interventions and sleep interventions not included in the review

The current review only included interventions which were described as specifically targeting sleep. Some of the interventions included (e.g., mindfulness intervention, seltorexant, prazosin) can also, or even primarily, be used to target other (mental) health symptoms such as anxiety, depression, or high blood pressure. Studies examining such interventions to target (mental) health conditions other than disturbed sleep were not included in the current review. As such, the current findings should not be interpreted as reflecting the effect or safety of an intervention outside the context of sleep. It is notable that no studies on some common pharmacological sleep interventions such as benzodiazepines were identified in the current review, despite their association with suicidal behavior [e.g., 91]. Similarly, a number of non-pharmacological sleep interventions [22,112], such as behavior change methods (e.g., parents setting up scheduled bedtimes for children), physical exercise interventions (e.g., aerobic exercises) or listening to music, were not identified by the current review. It is possible that RCTs of such interventions did not assess suicidality and/or were examined outside the context of sleep (e.g., benzodiazepines being examined in the context of anxiety disorders). In addition, studies that did monitor suicidality but did not report on it (e.g., because no suicidal events occurred or because the study was terminated when suicidal events occurred), would be missed by systematic literature searches such as the current one. The reliance on active reporting of suicidal adverse events thus constitutes a selection bias of the current review. With regard to environmental/chronotherapeutic sleep interventions, we included four studies (2 efficacy studies, 2 safety studies) that used light exposure in the current review. Due to the low number of studies, no firm conclusions could be drawn about the effect or safety of light interventions on suicidality.

### 4.3 Treatment implications and directions for future research

In sum, the current review indicates that sleep interventions, particularly psychotherapeutic sleep interventions, not only improve sleep but can additionally reduce suicidal ideation. Most of the included study samples contained individuals with (sub)clinical levels of insomnia and/or depression. Next to that, other samples included participants with various other sleep disturbances (nightmares, shift work disorder, obstructive sleep apnea, narcolepsy) and/or other mental health conditions (PTSD/trauma, psychotic disorder, ADHD). As such, the findings primarily have implications for people with insomnia and/or depression but may also apply to individuals with other sleep disturbances or psychological conditions. As discussed earlier, CBT-I may be a potentially promising psychotherapeutic intervention to effectively reduce suicidality with comorbid insomnia. CBT-I is the first-line treatment for insomnia [113] and some initial findings suggest that it could potentially affect other sleep issues such as nightmares [114,115] and fear of sleep [116]. There are several training programs for CBT-I available to health care professionals [117]. CBT-I programs with direct contact between the health care professional and patient (in individual, group, or digital formats) are more effective in improving insomnia than self-help CBT-I programs [118]. Clinicians are recommended to discuss CBT-I format preferences with their patients [119] and closely monitor treatment adherence when digital CBT-I formats are utilized [45,117,120]. In people with mental health comorbidities (e.g., depression, PTSD), CBT-I should not be used as replacement of but rather as an add-on to disorder-specific treatment [102].

Even though we did not find that medications for sleep disorders increase the risk for suicidality, the possibility of suicidal adverse effects cannot be excluded due to the high uncertainty of evidence. As a result, medications targeting sleep disorders should be prescribed with caution, particularly in people with high levels of suicidality. Research indicates that the prescription and use of multiple medications, a phenomenon termed ‘polypharmacy’ [121], is common in people with suicidality/depression [122,123]. Polypharmacy is associated with detrimental outcomes, including overdose and suicidal behavior [124,125]. As such, it is critical to pay close attention to concurrent medication use, particularly in people with multiple comorbidities. In case pharmacotherapy is deemed necessary (e.g., to target severe sleeplessness in the context of a crisis situation), health care professionals are recommended to prescribe these medications only for short periods of time and to closely monitor suicidal ideation and behavior.

In order to draw more definitive conclusions and refine treatment implications, more research is needed to tackle the limitations of the existing literature. Overall, there is currently a limited number of high quality RCTs on the effect and safety of different types of sleep interventions on suicidality. As such, the reliance on small studies with large error margins decreased the certainty in the observed overall effect and, due to deflated heterogeneity estimates, prevented examining the association between suicidality and sleep effect sizes. Moreover, we observed large inconsistencies in practices of monitoring and reporting suicidality as adverse effects, and suicidality being used as an exclusion criterion for study participation, particularly in the safety studies. Most efficacy studies relied on suicidal ideation as a measure of suicidality, and only a few examined suicidality at follow-up assessments [e.g., 43,46]. Some studies used unvalidated and/or single-item assessments of suicidality. Consequently, more large and high quality RCTs are needed to investigate the (long-term) effect and safety of different types of sleep interventions on suicidal thoughts and behavior (assessed with validated instruments), particularly in people with elevated levels of suicidality. More high quality RCTs across various sleep disturbances and/or psychological conditions would allow for more targeted reviews and/or analyses (e.g., meta-regressions; moderator analyses) and thus for drawing more refined treatment recommendations in specific patient populations. Importantly, we strongly recommend adherence to guidelines for monitoring and reporting adverse effects [126]. During the literature search of the current review, we came across several promising study protocols, such as the TAILOR study protocol (ClinicalTrials.gov ID: NCT05390918) and a protocol of a sleep study in veterans (ClinicalTrials.gov ID: NCT03603717). This suggests that it may soon be possible to draw firmer conclusions on the effect and safety of different types of sleep interventions on suicidality.

### 4.4 Conclusion

Taken together, this review provides support for the positive effects of sleep interventions on reducing suicidal ideation, primarily in people with insomnia and/or depression. Based on the results, we recommend health care professionals to utilize psychotherapeutic sleep interventions such as CBT-I, to reduce suicidal ideation in the context of disturbed sleep. Due to the uncertainty of evidence surrounding the effect and safety of pharmacological sleep interventions on suicidality, health care professionals are recommended to closely monitor suicidality when prescribing medications targeting sleep disturbance.

## Funding

This work was supported by the Ministry of Health, Welfare and Sport. The funders of the study had no role in study design, data collection, data analysis, data interpretation, or writing of the report.

## CRediT authorship contribution statement

**Paula von Spreckelsen:** Writing – review & editing, Writing – original draft, Visualization, Software, Methodology, Formal analysis, Data curation, Conceptualization. **Daan Schouten:** Writing – review & editing, Writing – original draft, Methodology, Formal analysis, Data curation, Conceptualization. **Natasha G. Waslam:** Writing – review & editing, Data curation. **Patricia Katuin:** Writing – review & editing, Conceptualization. **Henriette D. Heering:** Writing – review & editing, Supervision. **Caroline Planting:** Writing – review & editing, Data curation. **Niki Antypa:** Writing – review & editing, Validation. **Liia Kivelä:** Writing – review & editing, Validation. **Marike Lancel:** Writing – review & editing, Supervision, Conceptualization. **Lizanne J.S. Schweren:** Writing – review & editing, Supervision, Resources, Project administration, Methodology, Conceptualization.

## Declaration of competing interest

None of the authors declare competing interests.

## Figures and Tables

**Table:** 

Database searched	Platform	Years of coverage	Records	Records after duplicates removed
PubMed	PubMed	1946 – Present	644	644
Embase	Embase.com	1971 - Present	3.680	3.232
APA PsycInfo	EBSCO	1968 - Present	409	121
Web of Science	Clarivate	1900 - Present	662	175
Cochrane CENTRAL	Wiley	1992 - Present	525	540
Scopus	Elsevier	1970 - Present	2.556	325
**Total**			**8.476**	**5.037**

Search	PubMed	Results
	Query	
#4	Search: #1 AND #2 AND #3 Sort by: Most Recent	644
#3	Search: “Randomized Controlled Trial" [pt] OR “Controlled Clinical Trial" [pt] OR “Randomized Controlled Trials as Topic" [Mesh] OR “Random allocation" [Mesh] OR “Double-blind method" [Mesh] OR “Single-blind method" [Mesh] OR “control groups" [Mesh] OR “Clinical Trials as Topic" [Mesh] OR “Placebos" [Mesh] OR random [tw] OR randomly [tw] OR randomised [tw] OR randomized [tw] OR randomizing [tw] OR randomizing [tw] OR placebo [tiab] OR ((singl∗[tw] OR doubl∗[tw] OR trebl∗[tw] OR tripl∗[tw]) AND (mask∗[tw] OR blind∗[tw] OR dumm∗[tw])) OR RCT [tiab] OR RCTs [tiab] OR “clinical trial∗" [tiab] OR “controlled trial∗" [tiab] OR crossover∗[tiab] OR “cross-over∗" [tiab] Sort by: Most Recent	2,513,327
#2	Search: “Sleep" [Mesh] OR “Sleep Wake Disorders" [Mesh] OR sleep∗[tiab] OR “sleep disorder∗" [tiab] OR “sleep wake disorder∗" [tiab] OR “sleep initiation" [tiab] OR “sleep onset" [tiab] OR “sleep latency" [tiab] OR “sleep deprivation" [tiab] OR “sleep quality" [tiab] OR “sleep hygiene" [tiab] OR “sleep disturbance∗" [tiab] OR “sleep problem∗" [tiab] OR “sleep maintenance" [tiab] OR “sleep fragmentation∗" [tiab] OR “sleep duration∗" [tiab] OR insomni∗[tiab] OR “insomnia disorder∗" [tiab] OR hypersomnolen∗[tiab] OR “hypersomnolence disorder∗" [tiab] OR narcolep∗[tiab] OR apnea∗[tiab] OR “obstructive sleep apnea∗" [tiab] OR “central sleep apnea∗" [tiab] OR “sleep-related hypoventilation" [tiab] OR “circadian rhythm∗" [tiab] OR “circadian rhythm sleep-wake disorder∗" [tiab] OR parasomni∗[tiab] OR “non-Rapid Eye Movement∗" [tiab] OR “non-REM∗" [tiab] OR “nonRapid Eye Movement∗" [tiab] OR nonREM∗[tiab] OR “non-Rapid Eye Movement sleep arousal disorder∗" [tiab] OR nightmare∗[tiab] OR “nightmare disorder∗" [tiab] OR “Rapid Eye Movement∗" [tiab] OR REM [tiab] OR “Rapid Eye Movement Sleep behavior disorder∗" [tiab] OR dream∗[tiab] OR nap [tiab] OR naps [tiab] OR bedtime∗[tiab] OR “bed time∗" [tiab] OR “night rest∗" [tiab] OR “time in bed" [tiab] OR “sleep time∗" [tiab] OR “night waking" [tiab] OR drowsiness [tiab] OR somnolen∗[tiab] OR hypersomni∗[tiab] OR “early awakening∗" [tiab] OR sleepwalking∗[tiab] OR “restless legs syndrome∗" [tiab] OR “restless leg syndrome∗" [tiab] OR “periodic limb movement disorder∗" [tiab] Sort by: Most Recent	382,875
#1	Search: “Suicide" [Mesh] OR suicid∗[tiab] OR “self-kill∗" [tiab] OR “self-poison∗" [tiab] OR “self-stabb∗" [tiab] OR parasuicid∗[tiab] Sort by: Most Recent	126,062

Key: [Mesh] = medical subject heading, [tiab] = title, abstract, author supplied keywords.

**Table:** 

No.	Embase (Embase.com)	Results
	Query	
**#5**	**#4** AND (**'article'**/it OR **‘article in press'**/it OR **‘chapter'**/it OR **‘preprint'**/it)	**3680**
**#4**	**#1** AND **#2** AND **#3**	**6409**
**#3**	**‘controlled clinical trial'**/de OR **‘randomized controlled trial'**/exp OR **‘randomized controlled trial topic'**/de OR **‘controlled clinical trial'**/exp OR **‘controlled study'**/exp OR **‘clinical trial topic'**/de OR **‘clinical trial'**/de OR **‘randomization'**/exp OR **‘double blind procedure'**/exp OR **‘single blind procedure'**/de OR **‘placebo'**/exp OR **‘crossover procedure'**/exp OR **‘crossover procedure'**:ab,ti,kw OR **‘double blind procedure'**:ab,ti,kw OR **‘randomized controlled trial'**:ab,ti,kw OR **‘single blind procedure'**:ab,ti,kw OR **‘placebo'**:ab,ti,kw OR **‘clinical trial'**:ab,ti,kw OR **random**:ab,ti,kw OR **randomly**:ab,ti,kw OR **randomised**:ab,ti,kw OR **randomized**:ab,ti,kw OR **randomizing**:ab,ti,kw OR **randomizing**:ab,ti,kw OR **factorial∗**:ab,ti,kw OR **crossover∗**:ab,ti,kw OR **‘cross-over∗'**:ab,ti,kw OR **placebo∗**:ab,ti,kw OR **rct**:ab,ti,kw OR **rcts**:ab,ti,kw OR ((**singl∗**:ab,ti,kw OR **doubl∗**:ab,ti,kw OR **trebl∗**:ab,ti,kw OR **tripl∗**:ab,ti,kw) AND (**mask∗**:ab,ti,kw OR **blind∗**:ab,ti,kw OR **dumm∗**:ab,ti,kw)) OR **‘clinical trial∗'**:ab,ti,kw OR **‘controlled trial∗'**:ab,ti,kw	**13,271,273**
**#2**	**‘sleep'**/exp OR **‘sleep disorder'**/exp OR **sleep∗**:ab,ti,kw OR **‘sleep disorder∗'**:ab,ti,kw OR **‘sleep wake disorder∗'**:ab,ti,kw OR **‘sleep initiation'**:ab,ti,kw OR **‘sleep onset'**:ab,ti,kw OR **‘sleep latency'**:ab,ti,kw OR **‘sleep deprivation'**:ab,ti,kw OR **‘sleep quality'**:ab,ti,kw OR **‘sleep hygiene'**:ab,ti,kw OR **‘sleep disturbance∗'**:ab,ti,kw OR **‘sleep problem∗'**:ab,ti,kw OR **‘sleep maintenance'**:ab,ti,kw OR **‘sleep fragmentation∗'**:ab,ti,kw OR **‘sleep duration∗'**:ab,ti,kw OR **insomni∗**:ab,ti,kw OR **‘insomnia disorder∗'**:ab,ti,kw OR **hypersomnolen∗**:ab,ti,kw OR **‘hypersomnolence disorder∗'**:ab,ti,kw OR **narcolep∗**:ab,ti,kw OR **apnea∗**:ab,ti,kw OR **‘obstructive sleep apnea∗'**:ab,ti,kw OR **‘central sleep apnea∗'**:ab,ti,kw OR **‘sleep-related hypoventilation'**:ab,ti,kw OR **‘circadian rhythm∗'**:ab,ti,kw OR **‘circadian rhythm sleep-wake disorder∗'**:ab,ti,kw OR **parasomni∗**:ab,ti,kw OR **‘non-rapid eye movement∗'**:ab,ti,kw OR **‘non-rem∗'**:ab,ti,kw OR **‘nonrapid eye movement∗'**:ab,ti,kw OR **nonrem∗**:ab,ti,kw OR **‘non-rapid eye movement sleep arousal disorder∗'**:ab,ti,kw OR **nightmare∗**:ab,ti,kw OR **‘nightmare disorder∗'**:ab,ti,kw OR **‘rapid eye movement∗'**:ab,ti,kw OR **rem**:ab,ti,kw OR **‘rapid eye movement sleep behavior disorder∗'**:ab,ti,kw OR **dream∗**:ab,ti,kw OR **nap**:ab,ti,kw OR **naps**:ab,ti,kw OR **bedtime∗**:ab,ti,kw OR **‘bed time∗'**:ab,ti,kw OR **‘night rest∗'**:ab,ti,kw OR **‘time in bed'**:ab,ti,kw OR **‘sleep time∗'**:ab,ti,kw OR **drowsiness**:ab,ti,kw OR **somnolen∗**:ab,ti,kw OR **hypersomni∗**:ab,ti,kw OR **‘early awakening∗'**:ab,ti,kw OR **‘night waking∗'**:ab,ti,kw OR **sleepwalking∗**:ab,ti,kw OR **‘restless leg∗ syndrome∗'**:ab,ti,kw OR **‘periodic limb movement disorder∗'**:ab,ti,kw	**694,782**
**#1**	**‘suicide'**/exp OR **‘suicidal behavior'**/exp OR **‘suicidal behavior'**:ab,ti,kw OR **suicid∗**:ab,ti,kw OR **‘self-kill∗'**:ab,ti,kw OR **‘self-poison∗'**:ab,ti,kw OR **‘self-stab∗'**:ab,ti,kw	**180,823**

Key: Ab, ti, kw searches in abstract, title and author supplied keywords,/exp searches Emtree preferred indexing term, it = publication type

**Table:** 

#	APA PsycInfo (EBSCO)	Results
	Query	
S5	S1 AND S2 AND S3 – Limiters Academic journals	409
S4	S1 AND S2 AND S3	436
S3	DE “Treatment Effectiveness Evaluation” OR DE “Mental Health Program Evaluation” OR DE “Placebo” OR DE “Clinical Trials” OR DE “Randomized Clinical Trials” OR DE “Experimental Design” OR DE “Randomized Controlled Trials” OR DE “Experiment Controls” OR DE “Random Sampling” OR TI (placebo∗ OR random OR randomly OR randomised OR randomized OR randomizing OR randomizing OR factorial∗ OR ((singl∗ OR doubl∗ OR trebl∗ OR tripl∗) N3 (blind∗ OR mask∗ OR dummy)) OR (control∗ N3 trial∗) OR allocat∗ OR assign∗ OR crossover∗ OR “cross over∗" OR RCT OR RCTs OR “clinical trial∗” OR “controlled trial∗“) OR AB (placebo∗ OR random OR randomly OR randomised OR randomized OR randomizing OR randomizing OR factorial∗ OR ((singl∗ OR doubl∗ OR trebl∗ OR tripl∗) N3 (blind∗ OR mask∗ OR dummy)) OR (control∗ N3 trial∗) OR allocat∗ OR assign∗ OR crossover∗ OR “cross over∗" OR RCT OR RCTs OR “clinical trial∗” OR “controlled trial∗“) OR KW((placebo∗ OR random OR randomly OR randomised OR randomized OR randomizing OR randomizing OR factorial∗ OR ((singl∗ OR doubl∗ OR trebl∗ OR tripl∗) N3 (blind∗ OR mask∗ OR dummy)) OR (control∗ N3 trial∗) OR allocat∗ OR assign∗ OR crossover∗ OR “cross over∗" OR RCT OR RCTs OR “clinical trial∗” OR “controlled trial∗“) OR MR (“clinical trial”)	477,452
S2	DE “Dreaming” OR DE “Napping” OR DE “NREM Sleep” OR DE “REM Sleep” OR DE “Sleepiness” OR DE “Sleepwalking” OR DE “Sleep” OR DE “REM Sleep” OR DE “Sleep Onset” OR DE “Sleep Quality” OR DE “Sleep Apnea” OR DE “Sleep-Related Hypoventilation” OR DE “Sleep Arousal Disorders” OR DE “Sleep Terrors” OR DE “Sleep Deprivation” OR DE “Sleep Wake Cycle” OR DE “Sleep Wake Disorders” OR DE “Hypersomnia” OR DE “Insomnia” OR DE “Narcolepsy” OR DE “Parasomnias” OR DE “Sleep Apnea” OR DE “Nightmares” OR DE “REM Sleep Behavior Disorder” OR DE “REM Dream Deprivation” OR DE “REM Sleep” DE “Restless Leg Syndrome” OR TI (sleep∗ OR “sleep disorder∗” OR “sleep wake disorder∗” OR “sleep initiation” OR “sleep onset” OR “sleep latency” OR “sleep deprivation” OR “sleep quality” OR “sleep hygiene” OR “sleep disturbance∗” OR “sleep problem∗” OR “sleep maintenance” OR “sleep fragmentation∗” OR “sleep duration∗” OR insomni∗ OR “insomnia disorder∗” OR hypersomnolen∗ OR “hypersomnolence disorder∗” OR narcolep∗ OR apnea∗ OR “obstructive sleep apnea∗” OR “central sleep apnea∗” OR “sleep-related hypoventilation” OR “circadian rhythm∗” OR “circadian rhythm sleep-wake disorder∗” OR parasomni∗ OR “non-Rapid Eye Movement∗” OR “non-REM∗” OR “nonRapid Eye Movement∗” OR nonREM∗ OR “non-Rapid Eye Movement sleep arousal disorder∗” OR nightmare∗ OR “nightmare disorder∗” OR “Rapid Eye Movement∗” OR REM OR “Rapid Eye Movement Sleep behavior disorder∗” OR dream∗ OR nap OR naps OR bedtime∗ OR “bed time∗” OR “night rest∗” OR “time in bed” OR “sleep time∗” OR drowsiness∗ OR somnolen∗ OR hypersomni∗ OR “early awakening∗” OR “night waking” OR sleepwalking OR “Restless Leg∗ Syndrome∗" OR “periodic limb movement disorder∗“) OR AB (sleep∗ OR “sleep disorder∗” OR “sleep wake disorder∗” OR “sleep initiation” OR “sleep onset” OR “sleep latency” OR “sleep deprivation” OR “sleep quality” OR “sleep hygiene” OR “sleep disturbance∗” OR “sleep problem∗” OR “sleep maintenance” OR “sleep fragmentation∗” OR “sleep duration∗” OR insomni∗ OR “insomnia disorder∗” OR hypersomnolen∗ OR “hypersomnolence disorder∗” OR narcolep∗ OR apnea∗ OR “obstructive sleep apnea∗” OR “central sleep apnea∗” OR “sleep-related hypoventilation” OR “circadian rhythm∗” OR “circadian rhythm sleep-wake disorder∗” OR parasomni∗ OR “non-Rapid Eye Movement∗” OR “non-REM∗” OR “nonRapid Eye Movement∗” OR nonREM∗ OR “non-Rapid Eye Movement sleep arousal disorder∗” OR nightmare∗ OR “nightmare disorder∗” OR “Rapid Eye Movement∗” OR REM OR “Rapid Eye Movement Sleep behavior disorder∗” OR dream∗ OR nap OR naps OR bedtime∗ OR “bed time∗” OR “night rest∗” OR “time in bed” OR “sleep time∗” OR drowsiness OR somnolen∗ OR hypersomni∗ OR “early awakening∗” OR “night waking” OR sleepwalking OR “Restless Leg∗ Syndrome∗" OR “periodic limb movement disorder∗“) OR KW(sleep∗ OR “sleep disorder∗” OR “sleep wake disorder∗” OR “sleep-wake disorder∗” OR “sleep initiation” OR “sleep onset” OR “sleep latency” OR “sleep deprivation” OR “sleep quality” OR “sleep hygiene” OR “sleep disturbance∗” OR “sleep problem∗” OR “sleep maintenance” OR “sleep fragmentation∗” OR “sleep duration∗” OR insomni∗ OR “insomnia disorder∗” OR hypersomnolen∗ OR “hypersomnolence disorder” OR narcolep∗ OR apnea∗ OR “obstructive sleep apnea∗” OR “central sleep apnea∗” OR “sleep-related hypoventilation” OR “circadian rhythm∗” OR “circadian rhythm sleep-wake disorder∗” OR parasomni∗ OR “non-Rapid Eye Movement∗” OR “non-REM∗” OR “nonRapid Eye Movement∗” OR nonREM∗ OR “non-Rapid Eye Movement sleep arousal disorder∗” OR nightmare∗ OR “nightmare disorder∗” OR “Rapid Eye Movement∗” OR REM OR “Rapid Eye Movement Sleep behavior disorder∗” OR dream∗ OR nap OR naps OR bedtime∗ OR “bed time∗” OR “night rest∗” OR “time in bed” OR “sleep time∗” OR drowsiness OR somnolen∗ OR hypersomni∗ OR “early awakening∗” OR “night waking” OR sleepwalking OR “Restless Leg∗ Syndrome∗" OR “periodic limb movement disorder∗“)	148,200
S1	DE “Suicide” OR DE “Attempted Suicide” OR DE “Suicidality” OR DE “Suicidal Ideation” OR DE “Suicide Prevention” OR DE “Suicidology” OR DE “Self-Poisoning” OR DE “Military Suicide” OR DE “Youth Suicide” OR TI (suicid∗ OR “self-kill∗” OR “self-poison∗” OR “self-stabb∗” OR parasuicid∗) OR AB (suicid∗ OR “self-kill∗” OR “self-poison∗” OR “self-stabb∗” OR parasuicid∗) OR KW(suicid∗ OR “self-kill∗” OR “self-poison∗” OR “self-stabb∗” OR parasuicid∗)	86,285

Key: DE = Descriptors,TI = title, AB = abstract and KW= Searches for keywords in the uncontrolled content description of the document

**Table:** 

	**Web of Science (Core collection) – Clarivate** - WOS.SCI: 1900 to 2025 - WOS.AHCI: 1975 to 2025 - WOS.ESCI: 2005 to 2025 - WOS.SSCI: 1956 to 2025	
**Nr.**	**Query**	Results
#5	Search: #3 AND #2 AND #1 and Article or Early Access or Book Chapters (Document Types)	662
#4	Search: #3 AND #2 AND #1	864
#3	Search: TS=(placebo∗ OR random OR randomly OR randomised OR randomized OR randomizing OR randomizing OR factorial∗ OR ((singl∗ OR doubl∗ OR trebl∗ OR tripl∗) N3 (blind∗ OR mask∗ OR dummy)) OR (control∗ N3 trial∗) OR allocat∗ OR assign∗ OR crossover∗ OR “cross over∗" OR RCT OR RCTs OR “clinical trial∗” OR “controlled trial∗” OR “double-blind”)	4,154,060
#2	Search: TS=(sleep∗ OR “sleep disorder∗” OR “sleep wake disorder∗” OR “sleep initiation” OR “sleep onset” OR “sleep latency” OR “sleep deprivation” OR “sleep quality” OR “sleep hygiene” OR “sleep disturbance∗” OR “sleep problem∗” OR “sleep maintenance” OR “sleep fragmentation∗” OR “sleep duration∗” OR insomni∗ OR “insomnia disorder∗” OR hypersomnolen∗ OR “hypersomnolence disorder∗” OR narcolep∗ OR apnea∗ OR “obstructive sleep apnea∗” OR “central sleep apnea∗” OR “sleep-related hypoventilation” OR “circadian rhythm∗” OR “circadian rhythm sleep-wake disorder∗” OR parasomni∗ OR “non-Rapid Eye Movement∗” OR “non-REM∗” OR “nonRapid Eye Movement∗” OR nonREM∗ OR “non-Rapid Eye Movement sleep arousal disorder∗” OR nightmare∗ OR “nightmare disorder∗” OR “Rapid Eye Movement∗” OR REM OR “Rapid Eye Movement Sleep behavior disorder∗” OR dream∗ OR nap OR naps OR bedtime∗ OR “bed time∗” OR “night rest∗” OR “time in bed” OR “sleep time∗” OR drowsiness OR somnolen∗ OR hypersomni∗ OR “early awakening∗” OR “night waking” OR sleepwalking OR “Restless Leg∗ Syndrome∗" OR “periodic limb movement disorder∗“)	543,127
#1	Search: TS=(suicid∗ OR “self-kill∗” OR “self-poison∗” OR “self-stabb∗“)	147,335

Key: TS = topic, which includes title, abstract, author keywords and Web of Science Keywords Plus./TI = title, AK = author keywords

**Table:** 

ID	Cochrane CENTRAL (Wiley)	Hits
	Search	
#1	(suicid∗ OR self-killing∗ OR self-poisoning∗ OR self-stabbing∗):ab,ti,kw	9300
#2	(sleep∗ OR “sleep disorder” OR “sleep disorders” OR “sleep wake disorder” OR “sleep wake disorders” OR “sleep initiation” OR “sleep onset” OR “sleep latency” OR “sleep deprivation” OR “sleep quality” OR “sleep hygiene” OR “sleep disturbance” OR “sleep disturbances” OR “sleep problem” OR “sleep problems” OR “sleep maintenance” OR “sleep fragmentation” OR “sleep fragmentations” OR “sleep duration” OR “sleep durations” OR insomni∗ OR “insomnia disorder” OR “insomnia disorders” OR hypersomnolen∗ OR “hypersomnolence disorder” OR “hypersomnolence disorders” OR narcolep∗ OR apnea∗ OR “obstructive sleep apnea” OR “obstructive sleep apneas” OR “central sleep apnea” OR “central sleep apneas” OR “sleep related hypoventilation” OR “circadian rhythm” OR “circadian rhythm sleep wake disorder” OR “circadian rhythm sleep wake disorders” OR parasomni∗ OR “non Rapid Eye Movement” OR “non Rapid Eye Movement sleep arousal disorder” OR “non Rapid Eye Movement sleep arousal disorders” OR “non REM” OR “non REM sleep arousal disorder” OR “non REM sleep arousal disorders” OR “nonRapid Eye Movement” OR “nonRapid Eye Movement sleep arousal disorder” OR “nonRapid Eye Movement sleep arousal disorders” OR nonREM OR “nonREM sleep arousal disorder” OR “nonREM sleep arousal disorders” OR nightmare OR nightmares OR “nightmare disorder” OR “nightmare disorders” OR “Rapid Eye Movement” OR “Rapid Eye Movements” OR REM OR “Rapid Eye Movement Sleep behavior disorder” OR “Rapid Eye Movement Sleep behavior disorders” OR dream∗ OR nap OR naps OR bedtime∗ OR “bed time” OR “bed times” OR “night rest” OR “night rests” OR “time in bed” OR “sleep time” OR “sleep times” OR “night waking” OR “night wakings” OR drowsiness OR somnolen∗ OR hypersomni∗ OR “early awakening” OR “early awakenings” OR “night waking” OR sleepwalking OR “restless leg syndrome” OR “restless legs syndrome” OR “periodic limb movement disorder” OR “periodic limb movement disorders”):ab,ti,kw	86,085
#3	#1 AND #2	1489
#4	#3 AND CENTRAL	1468
#5	#4 AND (CT-gov OR ICTRP)	525

Key: ti,ab,kw searches in title, abstract and author supplied keywords

**Table:** 

History Count	Scopus (Elsevier)	Results
	Search	
#5	#1 AND #2 AND #3 AND (LIMIT-TO (DOCTYPE, “ar”) OR LIMIT-TO (DOCTYPE, “ch”))	2556
#4	#1 AND #2 AND #3	4222
#3	TITLE-ABS-KEY (placebo∗ OR random OR randomly OR randomised OR randomized OR randomizing OR randomizing OR factorial∗ OR ((singl∗ OR doubl∗ OR trebl∗ OR tripl∗) W/3 (blind∗ OR mask∗ OR dummy)) OR (control∗ W/3 trial∗) OR allocat∗ OR assign∗ OR crossover∗ OR “cross over∗" OR rct OR rcts OR “clinical trial∗" OR “controlled trial∗" OR “double-blind”)	6,369,87
#2	TITLE-ABS-KEY (sleep∗ OR “sleep disorder∗" OR “sleep wake disorder∗" OR “sleep initiation” OR “sleep onset” OR “sleep latency” OR “sleep deprivation” OR “sleep quality” OR “sleep hygiene” OR “sleep disturbance∗" OR “sleep problem∗" OR “sleep maintenance” OR “sleep fragmentation∗" OR “sleep duration∗" OR insomni∗ OR “insomnia disorder∗" OR hypersomnolen∗ OR “hypersomnolence disorder∗" OR narcolep∗ OR apnea∗ OR “obstructive sleep apnea∗" OR “central sleep apnea∗" OR “sleep-related hypoventilation” OR “circadian rhythm∗" OR “circadian rhythm sleep-wake disorder∗" OR parasomni∗ OR “non-Rapid Eye Movement∗" OR “non-REM∗" OR “nonRapid Eye Movement∗" OR nonrem∗ OR “non-Rapid Eye Movement sleep arousal disorder∗" OR nightmare∗ OR “nightmare disorder∗" OR “Rapid Eye Movement∗" OR rem OR “Rapid Eye Movement Sleep behavior disorder∗" OR dream∗ OR nap OR naps OR bedtime∗ OR “bed time∗" OR “night rest∗" OR “time in bed” OR “sleep time∗" OR drowsiness OR somnolen∗ OR hypersomni∗ OR “early awakening∗" OR “night waking” OR sleepwalking OR “Restless Leg∗ Syndrome∗" OR “periodic limb movement disorder∗")	787,533
#1	TITLE-ABS-KEY (suicid∗ OR “self-kill∗" OR “self-poison∗" OR “self-stabb∗")	199,789

Key: ABS, TITLE, AUTHKEY searches in abstract, title and author supplied keywords

**Figure:**
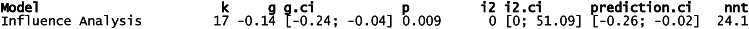
Influence analysis meta-analysis model on suicidal ideation as outcome

**Figure:**
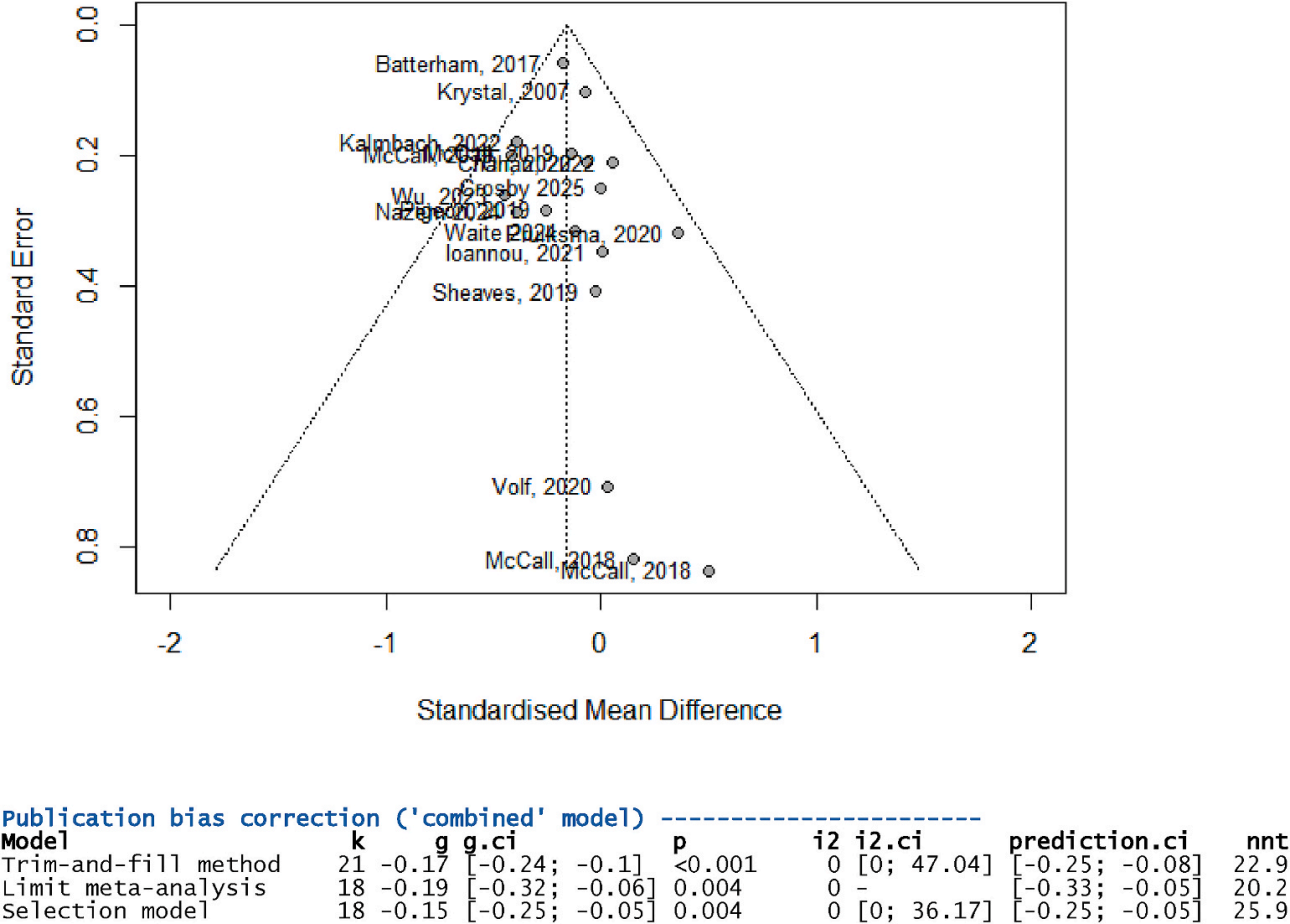
Funnel plot and publication bias correction results (outcome: suicidal ideation)

**Figure:**
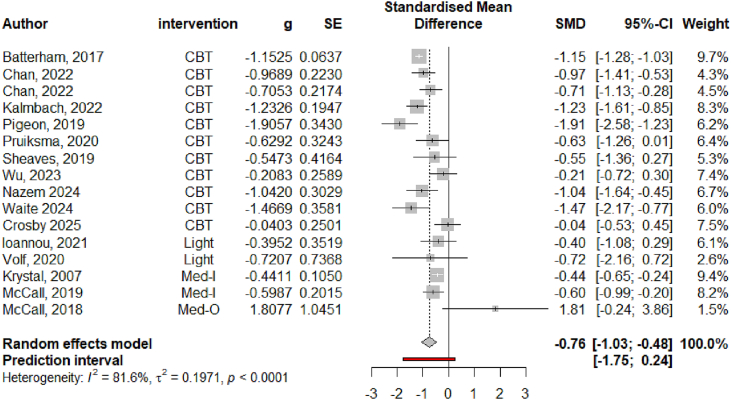
Forest Plot of all Efficacy Studies on sleep disturbance

**Figure:**
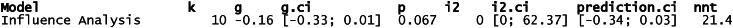
Influence analysis meta-analysis model on suicidal ideation as outcome

**Figure:**
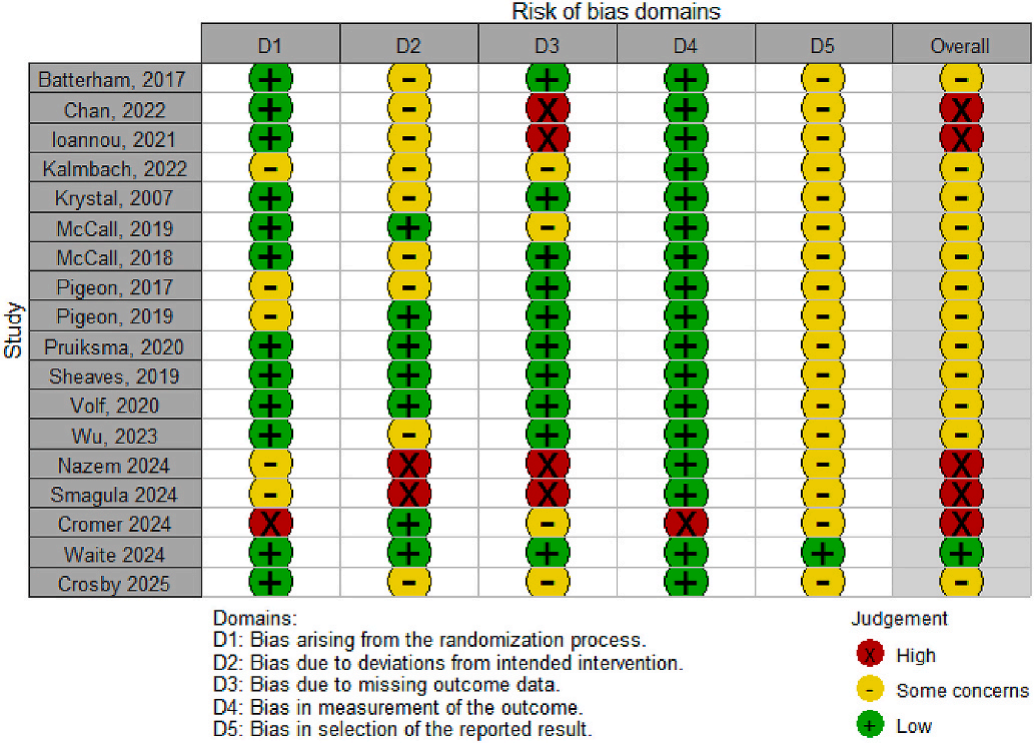
Risk of Bias Graph for all Efficacy Studies

**Figure:**
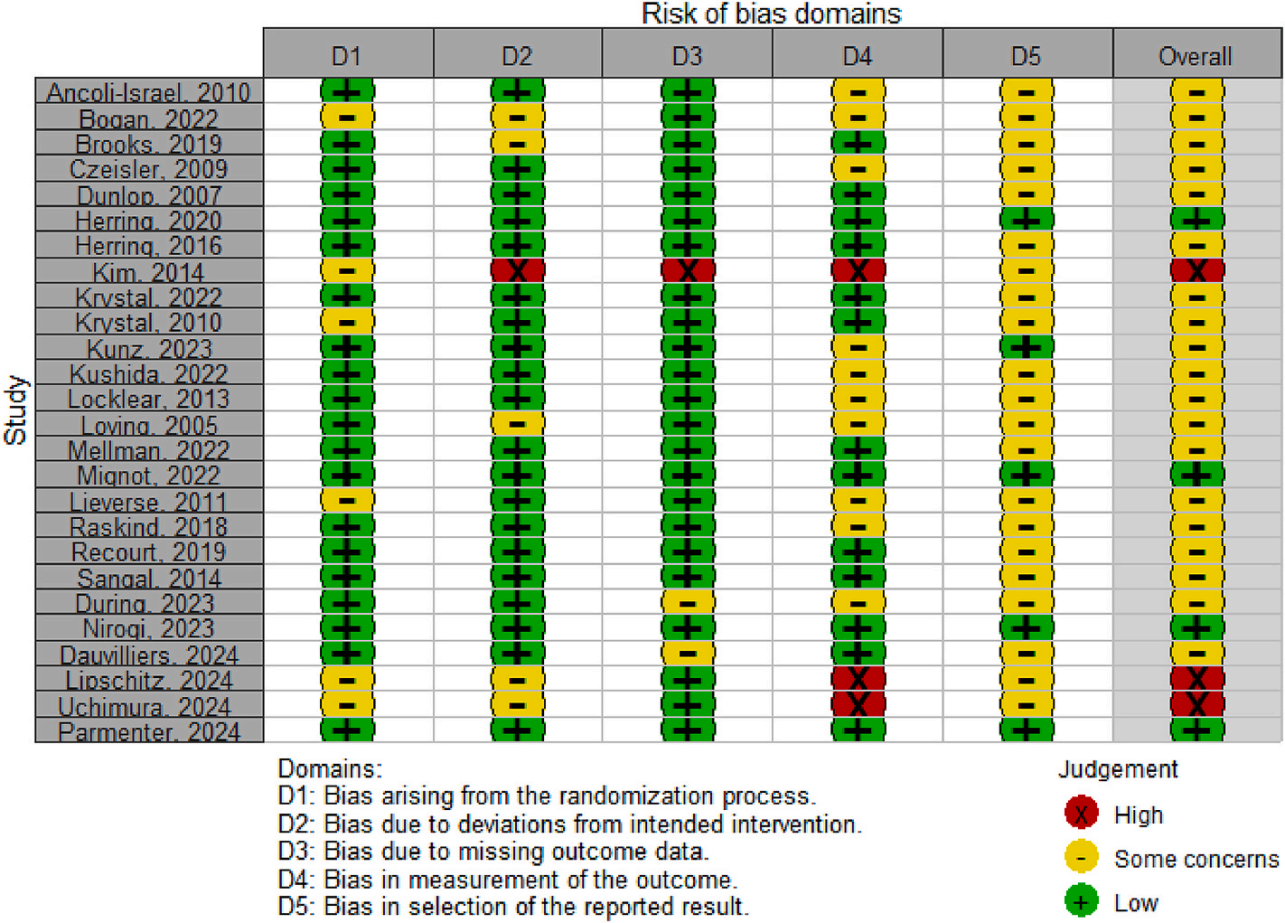
Risk of Bias Graph for all Safety Studies
